# Contextualizing the Impact of Snakebite Envenoming on Patients: A Qualitative Content Analysis of Patient-Specific Functional Scale Activities Using the International Classification of Functioning, Disability and Health

**DOI:** 10.3390/ijerph18189608

**Published:** 2021-09-12

**Authors:** Anna Tupetz, Ashley J. Phillips, Patrick E. Kelly, Loren K. Barcenas, Eric J. Lavonas, João Ricardo Nickenig Vissoci, Charles J. Gerardo

**Affiliations:** 1 Division of Emergency Medicine, Department of Surgery, Duke University School of Medicine, Durham, NC 27710, USA; anna.tupetz@duke.edu (A.T.); ashley.phillips@duke.edu (A.J.P.); patrick.kelly@duke.edu (P.E.K.); loren.barcenas@duke.edu (L.K.B.); jnv4@duke.edu (J.R.N.V.); 2 Department of Emergency Medicine, Denver Health and Hospital Authority, Denver, CO 80204, USA; eric.lavonas@dhha.org; 3 Duke Global Health Institute, Duke University, Durham, NC 27710, USA

**Keywords:** snake bite, PSFS, ICF, function, disability

## Abstract

To categorize the Patient-specific Functional Scale (PSFS) activities in snakebite envenoming (SBE) using the International Classification of Function (ICF) model in order to describe the impact of SBE on patients’ activities and daily lives and to develop a theoretical SBE model of functioning, we performed a post-hoc analysis of two multi-center, prospective studies, conducted at 14 clinical sites in the United States with consecutive SBE patients presenting to the emergency department. Qualitative content analysis and natural language processing were used to categorize activities reported in the PSFS using the ICF model. Our sample included 93 patients. The mean age was 43.0 (SD 17.9) years, most had lower extremity injuries (59%). A total of 99 unique activities representing eight domains came within the Activity and Participation component of the ICF model, with the majority in the Mobility and General Tasks and Demands domains. The main concerns of SBE patients are the ability to perform daily activities and to engage within their social environment. Applying the ICF model to SBE can facilitate the creation of a patient-centered treatment approach, moving beyond body-structural impairments towards a function-based treatment approach and facilitate early integration of rehabilitation services.

## 1. Introduction

Snakebite envenoming (SBE) is a global health concern and was designated a Category A Neglected Tropical Disease by the World Health Organization in 2017. This recognition offers access to new funding, research opportunities, potentially expands the availability of antivenom globally, and underscores the global burden of SBE [1]. SBE affects as many as 1.8 million people annually. While mortality is thought to be between 20,000–125,000 persons per year, an estimated 400,000 surviving patients are left living with permanent physical and psychological disabilities [2,3,4,5].

The physical complications of SBE have been studied; however, we have neglected how the physical complications such as tissue damage, pain and swelling [6] impacts the patient’s daily activities, their ability to generate income, return to recreational activities and emotional and mental well-being. Despite the significant disease burden and potentially detrimental impacts on the daily lives of the patients, there are no theoretical models to describe dysfunction in SBE populations. 

Currently available data on long-term morbidity and socioeconomic impact relies on non-specific disease models [7], missing the opportunity to best serve this particular patient population with a distinct array of functional impairments and possibly delaying early integration of rehabilitation services on a global scale.

The International Classification of Functioning, Disability and Health (ICF), developed by the World Health Organization, is one commonly used theoretical biopsychosocial model, that conceptualizes functionality as a product of disease, as well as environmental and personal factors [8,9,10]. This construct links the clinical signs and symptoms of disease with their impact on activities and participatory engagement, while incorporating contextual factors such as socio-economic determinants. SBE has not been assessed using this theoretical framework [11,12,13,14]. 

Previous studies use patient-centered outcomes to evaluate dysfunction and disability in the SBE population [6,7,15,16,17]. Patient reported outcomes (PRO), such as the Patient Specific Functional Scale (PSFS), can be used to fill this gap and inform a theoretical model for SBE [18]. The PSFS assesses dysfunction in daily tasks identified by the patients themselves and has already been validated for use in SBE clinical trials [7,19]. Due to its flexibility and contextual characteristics, the PSFS responses can be useful to qualitatively inform an SBE-specific dysfunction theoretical model, using the ICF [8,9]. Previously researchers have used PSFS responses to apply to the ICF model in musculoskeletal patient populations [20]. By definition, PROs are patient-centered outcomes; by allowing the patient to choose outcomes important to themselves, the PSFS provides data on clinical outcomes that patients value. They complement traditional clinical impairment-based outcome measures to better understand the patient’s level of dysfunction within their social context [20]. The PSFS and ICF can deepen our understanding of physical limitations, and how they impact daily activities and restrict participation. 

Our objective is to use the ICF to construct a theoretical model of SBE dysfunction by analyzing the content of the PSFS activities identified by SBE patients. We hypothesize that the patient reported activities will be largely varied across the domains of the ICF and could be explained further by the contextual factors of each participant. 

## 2. Materials and Methods

### 2.1. Study Design and Setting

We performed a post-hoc qualitative content analysis of the activities chosen by copperhead (*Agkistrodon contortrix*) snake envenomated patients in their functional assessment. The data stem from two multi-center, prospective studies conducted at 14 sites across the southeastern United States, where copperhead snakes are endemic. The first research project enrolled 20 patients in a prospective observational study assessing patient recovery following copperhead (*A.contortrix*) snakebite (NCT #01651299) [6]. The second study enrolled 74 patients in a randomized, double-blind clinical trial comparing Crotalidae polyvalent immune Fab (ovine) (CroFab®) antivenom (FabAV) (BTG International Inc, West Conshohocken, PA, USA) to placebo following *A. contortrix* snakebite (NCT 01864200) [15]. The parent studies have been approved by the relevant ethical bodies, and this presented analysis has been exempted from review by Duke Health IRB (Pro00103274). 

### 2.2. Participants

Eligible patients in the observational parent study were adults (18 years or older) who presented within 24 h with *A. contortrix* envenoming of a single extremity, distal to the elbow or knee. Exclusion criteria included prisoners, patients who were pregnant or breastfeeding, patients who were unable to read or comprehend the consent document or written assessment tools, patients who had a condition that would limit their ability to make a reliable self-report of functionality status, and patients who sustained a previous snake envenoming injury or injury to the affected limb within 30 days prior to enrollment [6]. Eligible patients in the randomized, controlled trial were 12 years or older who presented within 24 h with mild to moderate severity *A. contortrix* envenoming of one distal extremity. The same exclusion criteria applied with the additional exclusion criteria of severe envenoming in the prospective observational study. Severe envenoming was defined as swelling to the entire extremity, compartment syndrome, coagulopathy of possible medical importance, and/or more than minimal bleeding or hypotension.

### 2.3. Variables

Researchers collected information on sex, race, ethnicity, and envenoming location at baseline. The PSFS was first administered 3 days after SBE. The standardized PSFS protocol asks the patient to “identify three important activities that you are unable to do or are having difficulty with as a result of your snake bite.” Patients self-identified three activities, and then rated each time on an 11-point ordinal scale, where 0 is “unable to perform activity” and 10 is “able to perform the activity at the same level as before injury or problem.” For the parent studies, patients rated how much they were currently experiencing difficulty with the previously indicated activities using the same 11-point scale during follow up assessments at 7, 10, 14, 17, 21, 24 and 28 days. For this post-hoc analysis, we focus on the content and scope of activities chosen by the participants at the initial PSFS assessment, available from 93 patients. 

#### The International Classification of Functioning, Disability and Health (ICF)

The ICF model is the organizational and conceptual basis of this analysis. It conceptualizes functionality as a multidimensional interaction between the person’s Health Condition, Body Functions and Structures, Activities, Participation, Environmental Factors, and Personal Factors, defined as ICF components. In this analysis, given the specific question on activities using the PSFS, we focused on Activity and Participation components as reported by the participants. Activities are defined as the execution of individual-level functions; this concept includes the limitations or difficulty individuals experience in executing these functions. Participation is defined as an individual’s involvement in all of life’s interactions; like Activities, the concept of Participation includes consideration of restrictions or problems individuals experience. Each component is then further described using domains. The Activities and Participation components include 9 domains. Each domain then can further be specified by including second-level categories and third-level categories, that allow further descriptions of the tasks identified by participants.

Environmental and Personal Factors that serve as contextual components influence the disability experience. Environmental Factors are external physical and social elements which may serve as barriers or facilitators of the other ICF components. Personal Factors are individual-level characteristics (such as gender, age, and behavior patterns) of the person that may influence the disability experience [8]. 

### 2.4. Data Analysis

We used text mining techniques to pre-process the textual information collected through the PSFS, using the software R and the Latent Trait Models package. The process uses natural language processing techniques to remove word connectors, stop words, signs and punctuations as well as to reduce all text to lowercase, and lastly perform stemming and tokenization procedures to prepare the responses for the content analysis. We gathered unique occurrences for pre-processed activities into a data corpus that went through content analysis. Two coders, both with health-related Master of Science degrees and not involved in the data collection phase of the underlying research studies conducted the qualitative deductive content analysis. The first analyst is a rehabilitation professional and researcher with experience in applying the ICF model through prior clinical education as well as research experience, provided a one-time training in this specific analysis approach to the second analyst and introduced the structure of the ICF model utilizing the official guides provided by the WHO [10]. The training as well as coding procedures were overseen and approved by the principal investigator of the study as well as an expert in psychometric evaluations and qualitative research methods. The second analyst was trained in qualitative research methods including deductive content analysis, through didactical as well as applied research experience. The first analyst created two separate coding sheets, one for each of the coders with a list of the unique activities listed by the participants. The coding sheet followed the structure of the ICF and included the specific ICF codes as well as definitions. Each activity chosen by the participant was then analyzed and subsequently grouped into the official ICF codes by domain, chapter, ICF sub category 1 and ICF sub category 2, by using detailed definitions and descriptions of each code as provided by the WHO [10]. 

Using deductive content analysis, the first analyst coded each unique response to the PSFS into one or more domains of the ICF within the Activity and Participation components. The second analyst coded a subset of 30% to ensure agreement and validation of the coding process and discussed differences among the study team. No major discrepancies were observed in the coding process and agreement was reached between the coders. The coders continually discussed the coding and analysis with the research team to validate the coding scheme. If a reported activity fit more than one of the given classifications within the ICF, the analysts double coded the activities with a primary code, being the most specific one to describe the task, and secondary code that described the activity more broadly. For example, the analysts primarily coded the activity “buttoning a shirt” in the domain ‘Self-care’ (code d5) with the second level category ‘Dressing’ (d540), and ICF subcategory 2 ‘putting on clothes’ (d5400), and secondarily coded in the ‘Mobility’ domain (d4) describing ‘Fine hand use’(d440), and ‘Manipulating’ (d4402). We calculated the frequencies of each domain and second-level categories for each activity. In addition, we applied the entire ICF model to a case example, to illustrate how the ICF model can be used to characterize SBE specific dysfunction and its potential impact on the patient’s daily life.

## 3. Results

### 3.1. Participants

In total, we included data of 93 patients in the present study. One patient of the original studies had missing PSFS data at 3 days and was thus excluded for this manuscript. The sample included slightly more males (52%) and the majority (87%) was white. Nearly two-thirds (59%) of participants suffered lower extremity SBE (Table 1).

### 3.2. Patient Specific Functional Scale (PSFS) Activities According to the ICF

We classified a total of 99 unique patient-reported activities into eight of the nine Domains within the ICF Activities and Participation components. Mobility was the most frequently cited domain (n = 84). One domain in the ICF Activities and Participation components (Learning and Applying Knowledge) was not related to any activities identified by participants.

The analysts double coded the majority of unique activities (n = 83), assigning a primary and a secondary code. In order of frequency of unique occurrences, primary codes included Mobility (n = 23), Community, Social and Civic Life (n = 21), Self Care (n = 19), Domestic Life (n = 16), Major Life Areas (n = 12), Communication (n = 5) and General Tasks and Demands (n = 3). 

**Figure 1:**
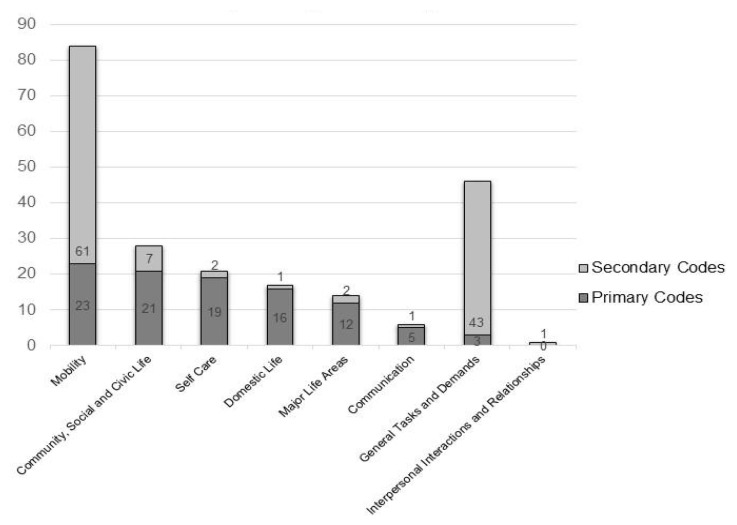
Frequency of unique activities per domain, stratified by primary and secondary codes.

Secondary codes included Mobility (n = 61); General Tasks and Demands (n = 43); Community, Social and Civic Life (n = 7); Self Care (n = 2) Major Life Areas (n = 2); Domestic Life (n = 1); Communication (n = 1); and Interpersonal Interactions and Relationships (n = 1). (Figure 1) 

**Figure 2:**
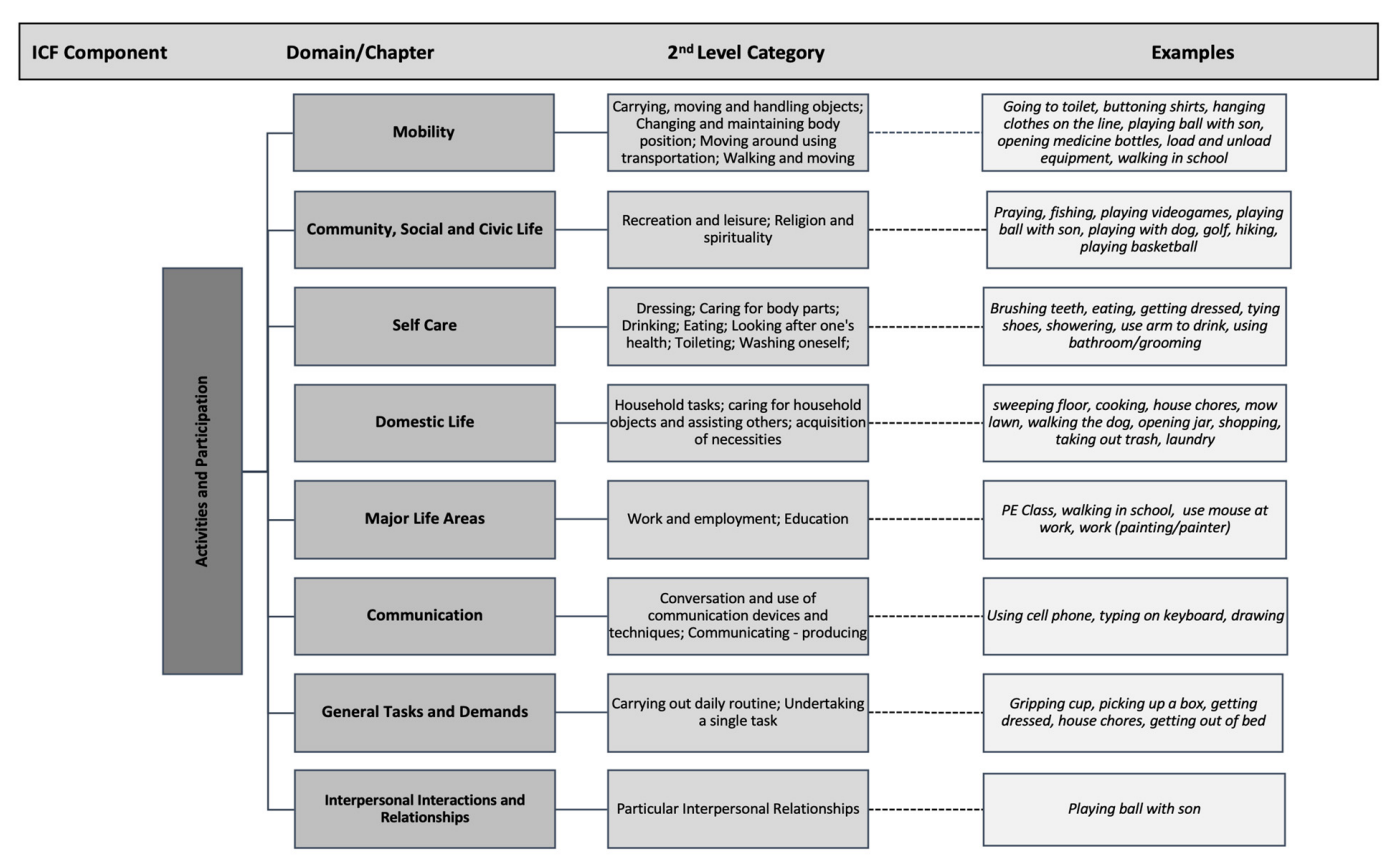
Overview of activities including examples coded in domains and 2nd-level categories of the ICF model.

Figure 2 provides a breakdown of domains as well as second-level categories that were used to classify the participant’s examples. In the Mobility Domain, participants named activities that fit into second-level categories such as “carrying, moving and handling objects; walking and moving; changing and maintaining body position”. In order to demonstrate the breadth of activities listed within the Mobility domain, being the most frequently coded section, we further specified this domain into third-level categories. 

**Figure 3:**
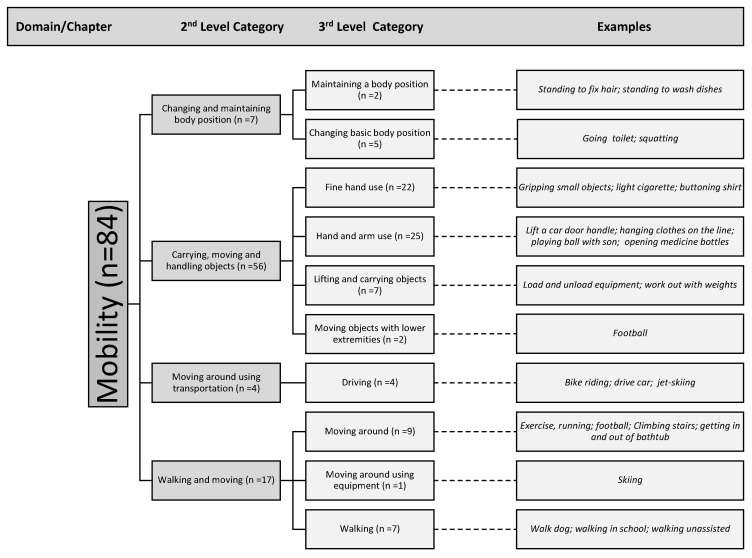
Overview of activities including examples coded within the Mobility domain, 2nd-level categories and 3rd-level categories.

Specific activities listed by the participants as seen in Figure 2 and Figure 3 include “playing ball with son, walking in school, opening medicine bottles” (Mobility Domain); “praying, fishing, yard work” (Community, Social and Civic Life); “using a cellphone, typing on a keyboard” (Communication) “construction work, walking in school” (Major Life Areas); “house chores, taking trash out, shopping” (Domestic Life) “showering, getting dressed, brushing teeth” (Self Care) The single participant response which indicated a code in the Interpersonal Interactions and Relationships domain was “playing ball with son”. 

Within the Mobility domain, the most coded section in the ICF shows that more than half of the mobility codes related to “carrying, moving and handling objects” (n = 56) This category represents a large variety in all aspects of daily life including the ability to open a car door, sport activities such as weightlifting, household chores, dressing, managing medications, work-related activities, and leisure activities with family members. (Figure 3).

### 3.3. Snakebite Envenoming (SBE)-Specific Translation of the ICF Model—Case Study

**Figure 4:**
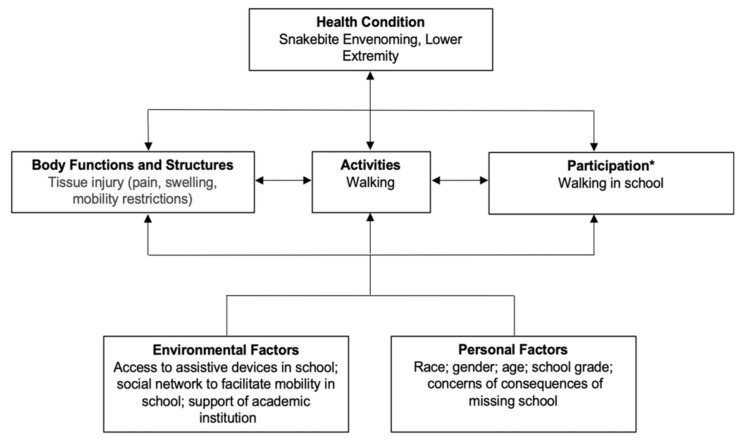
Case example application to International Classification of Functioning, Disability and Health (ICF) model; * listed activity by study participant.

Figure 4 depicts how the patient’s responses in the PSFS can be applied to the ICF framework and how each component of the model can relate to the next. This case study provides an example of assumptions made by the researchers about how one activity can be interpreted within the entirety of the ICF model. This patient reported “walking in school” as one of the three biggest difficulties he or she was experiencing since the SBE in their lower extremity (Health Condition). The SBE resulted in tissue injury that produced pain, swelling, and mobility restrictions (Body Function and Structures). Those signs and symptoms led to impairments in their walking ability (Activity) which impacted their school experience (Participation). Contextual factors within the ICF framework are Personal and Environmental factors. Personal factors include characteristics such as race, gender, age, and, in our specific case, what school grade they are in and their emotional status, which could include concerns about missing school. The environmental factors within the ICF are divided into the domains of products and technology; natural environment and human-made changes to the environment; support and relationships; attitudes; services, systems, and policies. Affecting this individual could include access to assistive devices within the school, the availability of a social network to facilitate mobility in school, as well as the level of support of the academic institution to accommodate the special needs of the student.

## 4. Discussion

This study informs a snakebite-specific theoretical model of dysfunction, presenting the breadth of impact that SBE has on most aspects of patient’s daily lives. In this qualitative analysis of the PSFS we have found that SBE patients are experiencing a variety of activity and participation limitations rather than solely physical impairments or mobility issues. By using the ICF model, we were able to identify and classify the domains which are most impacted in the patient’s daily lives after suffering SBE and suggest the value of having SBE-specific impairment models moving forward to elevate the standard of care for these patients. 

While most SBE patients share common physiological complications such as swelling and pain that explain the high frequency of codes in the Mobility domain, these symptoms can impact the patient’s lives very differently which leads to a high frequency of double coded activities in 8 of the 9 ICF domains. 

It is critical to pay attention to the social component and individual context of functional limitations, informing a quality-of-life theoretical model that is inclusive of contextual factors that are disease specific. To serve patients appropriately in their transition into the daily lives after SBE, the PSFS and ICF provide insight into the needs of this population and can inform best practices in SBE management, including rehabilitation services.

Current commonly used SBE outcome measures fall far short of adequately describing the impact of SBE on patient’s lives as they often include surrogate markers which are not patient-centered, assess only acute outcomes and not recovery, and are not comprehensive of the patient’s experience and/or not disease specific [17,21,22]. In a recent clinical trial of US antivenom, the primary efficacy outcome was coagulopathy (platelet count < 150,000/mm^3^, fibrinogen < 150 mg/dL) between completion of antivenom dosing and day 8 post envenomation [21]. The link between this laboratory value as an outcome measure and the more patient-centered outcome of clinical bleeding is not clearly defined, much less the impact of a bleeding episode on the patient’s function and quality of life.

Even the widely accepted and more comprehensive Snakebite Severity Score (SSS) used as an outcome measure primarily relies on either surrogate markers or components that are related to acute SBE [22]. For example, the Local Wound domain of the SSS uses the extent of pain, swelling, and ecchymosis of the affected extremity to determine severity of tissue injury in SBE. However, the relationship between the extent of swelling in SBE on subsequent function has not been clearly established [23]. This deficit is critically important as current SBE management guidelines recommend obtaining initial control based in part on these signs and symptoms in order to determine the need for additional antivenom [24]. Although we believe that the link exists between the acute findings of tissue injury and functional recovery, disability and quality of life (QoL), we now have a theoretical model that can be used to assess that relationship. There are several other generic outcome measures [25,26,27,28] which assess function and QoL combined. Unfortunately, these patient-reported outcome tools are not disease or dysfunction specific. Moreover, they are not specific enough to the individual patient to capture contextuality, which increases the risk of reporting bias [29]. A recent case report of a snake envenomation utilized the WHODAS 2.0 [27] to assess the extent of disability within its predefined domains, and while it provided rich insight by quantifying which domains are most affected by the envenomation, the tool does not put the limitations into context [30]. For example, generic survey tools that assess the distance a patient can walk are unable to capture the impact mobility restrictions have on the individual’s ability to care for themselves and their engagement with their community and maintaining their social life 

They also do not assess functional limitations in the context of the patient’s baseline performance. Gaining a better understanding of the impact SBE has on an individual’s QoL is critical in order to inform the potential risks and benefits of acute care treatments, and to also provide recommendations on follow up care for this patient population. Despite their limitations, these PRO and QoL measures do provide some important patient-centered information and can be used to validate our proposed SBE-specific dysfunction model moving forward [7].

### Limitations

In addition to the need to further assess internal validity, another limitation of this study is the need for external validation with other SBE populations to increase generalizability. This study population had predominantly mild severity, specificity of snake species (*A. contortrix*), and setting (US emergency departments). Validating the SBE dysfunction model in different geographic locations with higher snakebite burden, healthcare delivery systems, socioeconomic structures, other snake species and other antivenom treatment regimens, will be necessary to expand and refine the model. To expand the SBE dysfunction model, additional investigations linking biomarkers and clinical signs with the functional activities categorized within the ICF domains is needed. While the PSFS data allow the first insight into the breadth of activity and participation limitations, it provides limited data on contextual factors. Additional mixed methods studies to explore the contextual factors in SBE populations will be an integral part in further developing this model.

## 5. Conclusions

The main concerns of SBE patients are the ability to perform regular daily activities and to engage within their social environment, having a multifaceted impact on a variety of areas in their daily lives. The development of a theoretical function-based model, instead of symptom-based, can inform best practice interventions, that are patient centered and move beyond the immediate care in the Emergency Department and hospital. We believe that having an SBE specific dysfunction model can be used to inform treatment approaches, such as the role of early rehabilitation integration into the standard of care and thereby ultimately improve the care of patients on a global scale, tailored to the specific needs of the geographical location, healthcare delivery systems and snake species.

## Figures and Tables

**Table 1:** Participant characteristics.

Characteristic	Number	Percent
Total Enrolled	93	100%
Age, Mean years (SD)	43 (17.9)	-
Sex		
Male	48	52%
Female	45	48%
Race		
White	80	86%
Black or African-American	8	9%
Other	5	5%
Ethnicity		
Non-Hispanic	86	93%
Hispanic	7	7%
Envenoming Location		
Lower extremity	55	59%
Upper extremity	38	41%
Severity of Envenomation		
Mild	66	71%
Moderate	24	26%
Severe	3	3%
Initial PSFS Score, Median (IQR)	2.0 (0.3; 4.7)	-

## Data Availability

A list of unique codes analyzed in this manuscript are available in Appendix A.

## Abbreviations

Snakebite Envenomation (SBE); International Classification of Functioning, Disability and Health (ICF); Patient Specific Functional Scale (PSFS); Patient reported outcomes (PRO); Quality of Life (QoL); Snakebite Severity Score (SSS).

## Appendix A

Raw data and codes used for analysis post text mining techniques, PSFS activities.
